# Integrated EC-SERS Chip with Uniform Nanostructured EC-SERS Active Working Electrode for Rapid Detection of Uric Acid

**DOI:** 10.3390/s20247066

**Published:** 2020-12-10

**Authors:** Chu-Yu Huang, Hung-Che Hsiao

**Affiliations:** Department of Mechanical Engineering, National Chung Hsing University, Taichung 402, Taiwan; hungchr1225@gmail.com

**Keywords:** electrochemical surface-enhanced Raman spectroscopy, printed circuit board, uric acid, rapid detection, EC-SERS, nano imprint, hot embossing

## Abstract

Toxemia of pregnancy is a very dangerous disease for pregnant women. The mortality rate of toxemia of pregnancy is close to 10% to 15%. Early detection of pregnancy toxemia is to monitoring uric acid concentration in urine. The current mainstream method for detecting uric acid requires an enzyme (urate oxidase), which needs to be stored in a low-temperature environment, and the method requires complex chemical steps, which takes a longer time and more samples. In this study, we propose an integrated miniature three-electrode electrochemical surface-enhanced Raman spectroscopy chip (EC-SERS chip) suitable for rapid EC-SERS detection applications. The integrated microfluidic reservoir on the chip makes it easy to use, which is very suitable for rapid detection applications. The SERS active working electrode for the proposed integrated EC-SERS chip is a nanocone array polycarbonate (PC) substrate decorated with an evenly distributed and tightly packed array of gold nanospheres. It showed good uniformity and can be easily reproduced. The integrated EC-SERS chip is very small compared to the traditional electrochemical cell, which reduces the sample volume required for the testing. In addition, the chip is for one-time use only. It eliminates the need to clean electrochemical cells for reuse, thereby reducing the possibility of contamination and inaccurate detection. Various low-concentration Rhodamine 6G (R6G) solutions were tested to verify the performance of the developed EC-SERS chip. Experimental results showed that the proposed EC-SERS chip has a strong enhancement factor of up to 8.5 × 10^6^ and a very good EC-SERS uniformity (the relative standard deviation of EC-SERS intensity is as low as 1.41%). The EC-SERS chip developed has been further tested for the detection of uric acid in synthetic urine. The results showed that the EC-SERS signal intensity has a highly linear relationship with the logarithm of the uric acid concentration in synthetic urine, which indicates that the developed EC-SERS chip is suitable for the quantitative detection of uric acid in synthetic urine. Therefore, the developed EC-SERS chip is very promising to be used in routine and early diagnosis of pregnancy toxemia and may be used in many other medical tests, food safety, and biotechnology applications.

## 1. Introduction

In the human body, uric acid is the final product of purine metabolism. There is a lot of evidence that uric acid is related to certain metabolic diseases. Excessive uric acid in the blood can cause uric acid crystals to precipitate in joints and form arthritis, which can lead to gout. It can also damage the kidneys and cause chronic kidney disease or kidney stones. In pregnant women, it may cause pregnancy toxemia. Pregnancy toxemia is a hypertensive disease that occurs frequently in pregnant women, affecting 3% to 5% of pregnant women worldwide [1], and 10% to 15% of the deaths of pregnant women who have the disease are caused by pregnancy toxemia [2]. Because pregnancy toxemia is a heterogeneous disease, it is difficult to diagnose. The two main features in the pre-onset stage are new hypertension (systolic blood pressure ≥ 140 mm Hg or diastolic blood pressure ≥ 90 mm Hg) and a large amount of protein in the urine. However, it has been found that uric acid may be the cause of new-onset hypertensive symptoms in the early stage of pregnancy toxemia, and the symptoms of elevated uric acid usually appear earlier than hypertension and proteinuria [3,4]. When the concentration of uric acid in the patient’s urine is greater than 0.4 mM and with the symptoms of hypertension and proteinuria, this indicates that the patient is very likely to be related with pregnancy toxemia [5].

Currently, the main method for detecting uric acid requires the use of expensive urate oxidase to oxidize uric acid and produce allantoin and hydrogen peroxide. Hydrogen peroxide can react with 4-AAP and phenol (Phenol) to produce quinoneimine, which can be colorimetrically analyzed to determine its concentration [6,7]. However, this method requires the use of expensive enzymes, tedious chemical steps, longer test time, and more samples, which make the analysis method unsuitable for rapid and routine uric acid monitoring.

In the past decade, many works using SERS for uric acid detection [8,9,10,11,12]. For example, Ailin Wang et al. [13] using the Silver-carbon dots nanocomposites as SERS substrate to detect the SERS signal of oxidized tetramethylbenzidine (at 1605 cm^−1^). They found that the intensity of the SERS signal decreases when the concentration of UA ranges from 0.01 to 500 μM. However, the detection process involved a complex catalyzed oxidation and reduction. Melisew Tadele Alula et al. [14] used the Silver nanoparticles loaded magnetic nanospheres as SERS substrates to detection of uric acid in aqueous solution and urine sample. They find that the magnetic property of the substrate can help SERS measurements. They were able to achieve the limit of detection of 0.365 μM with a linear range of 0.5–10 μM. Gourab Bhattacharjee et al. [15] used hollow core-shell NPs to electrochemically sensing uric acid and ascorbic acid. Hollow core-shell NPs with a cubical shell showed a high SERS enhancement factor. Nevertheless, the uneven distribution of nanoparticle colloidal may result in uneven SERS enhancement over the sensing area. M. Pucetaite [10] et al. used a silver colloidal solution to titrate on alumina substrates for SERS detection of uric acid. The detection limit of uric acid in aqueous solutions was found to be 10^−6^ M. However, the use of nanoparticle colloidal as a SERS substrate will limit these methods of quantitatively detecting uric acid. Since the optical properties and the local electromagnetic field are affected by the metal nanoparticles’ size, shape, and distribution on the substrate. Irregular size, shape, and nonuniform distribution of nanoparticles will result in uneven SERS enhancement on such surfaces.

EC-SERS is a technology that combines electrochemistry and SERS. Recently, many analyses have shown that it greatly improves the sensitivity of SERS [16,17]. Brindersi et al. [18] showed that Fabric-based electrodes provide excellent EC-SERS signals, allowing rapid detection of levofloxacin at clinically relevant concentrations. It has also been shown that the label-free EC-SERS spectroscopy method may be a faster method for screening drugs from biological fluids [19]. Nanoscale gap junctions (called “hot spots”) between adjacent metal nanostructures are required for EC-SERS to produce strong sensitivity [20]. In many studies, EC-SERS also increases the adsorption of target molecules with low-affinity to SERS active substrates and therefore enhance the performance of biosensors; for example, it can enhance metabolite detection [18], antibiotic detection [21], and intravenous drug detection [22]. Lili Zhao et al. [23] used multilayered Au/Ag as EC-SERS substrate for the quantitative detection of uric acid. By a carefully controlled the nanoparticle drop coating process, they were able to obtain a multilayered deposition of Au/Ag nanoparticles. However, the process of multi-drop coating of nanoparticles is not easy to control, time-consuming, and difficult to reproduce, which may cause variations between substrates. Therefore, it is not conducive to the extensive use of routine analysis.

In this research, we propose an integrated EC-SERS chip that combines a three-electrode printed circuit board (PCB), an easy to reproduce uniform EC-SERS active working electrode, and an on-chip integrated microfluidic reservoir for quick detection of uric acid. Compared with the traditional three-electrode EC-SERS system and reaction cell, this integrated EC-SERS chip has the advantages of being convenient to carry, easy to use, and requiring less sample volume. Therefore, the integrated EC-SERS chip is very suitable for rapid EC-SERS detection applications. In addition, we propose a high uniformity and easy to reproduce EC-SERS active substrate for the EC-SERS working electrode. The EC-SERS active substrate can be batch-produced by hot embossing and sputtering deposition. The nanostructured EC-SERS active working electrode is a nanocone array PC substrate (moth-eye like structured PC substrate) decorated with an evenly distributed and tightly packed array of gold nanospheres. R6G solutions were used to test the performance of the developed EC-SERS chip, and the EC-SERS enhancement factor and EC-SERS signal uniformity were evaluated. The experimental results showed that the proposed EC-SERS active working electrode has a strong and uniform EC-SERS enhancement effect. The EC-SERS chip developed has been further tested to detect uric acid in synthetic urine. A good linear relationship between the EC-SERS signal intensity and the logarithm of the uric acid concentration in synthetic urine was observed. Therefore, the developed EC-SERS chip has the potential for routine and early diagnosis of pregnancy toxemia and may be used in various medical testing, food safety, and biotechnology applications.

## 2. Materials and Methods

### 2.1. Uniform Nanostructured Working Electrode Fabrication

**Figure 1:**
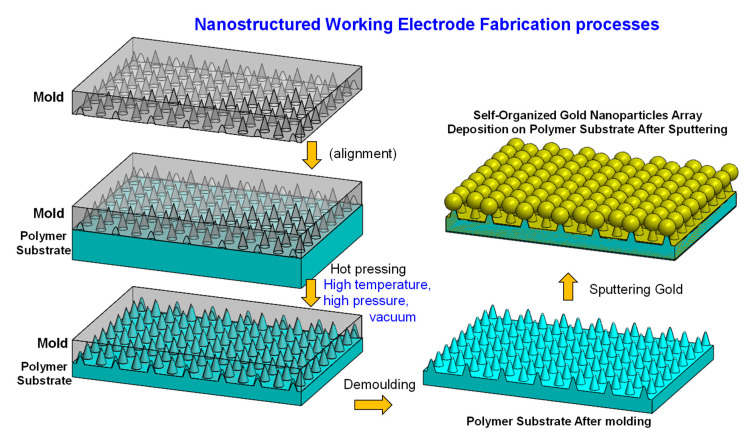
The fabrication process of the nanostructured polycarbonate (PC) substrate decorated with uniformly distributed and closely packed gold nanospheres is schematically illustrated here. The nanostructured nickel mold was heated and pressed on a PC plastic substrate for several minutes using a hot embossing machine. The PC substrate with nanocone array structure was released from the nickel mold after cool down to room temperature. A sputtering process was then used to form gold nanospheres at the tips of each nanocone.

For the uniform nanostructured Working Electrode, our research team has previously developed a SERS active substrate manufacturing technology [24], which produces a nanostructured PC substrate decorated with uniformly distributed and closely packed noble metal nanospheres. The SERS active substrate showed good uniformity and can be easily reproduced. In this study, we further utilized the technique to fabricate the SERS active working electrode for the integrated EC-SERS chip. Here, we briefly describe the fabrication process of a nanostructured PC substrate decorated with uniformly distributed and closely packed gold nanospheres. The manufacturing process is schematically illustrated in Figure 1. First, the structure of the aluminum substrate with a nano-conical cavity array was fabricated by using anodized aluminum self-assembly technology [25,26,27]. The manufacturing procedure of the nano-cone hole array on the aluminum substrate included electrolytic polishing, anodization, and anodized aluminum removal. In short, an aluminum substrate (99.999% pure) was electrolytically polished with an applied voltage of 25 V in a perchloric acid and absolute ethanol mixed solution (volume ratio of 1:3.5) for 2 min. The polished aluminum substrate was anodized in a 0.3 M oxalic acid solution at a voltage of 50 volts for 2 h. The substrate was then placed in a 5 wt% phosphoric acid solution at 35 °C for 1 h to remove the anodized aluminum oxide layer. To obtain the desired nano-cone hole array structure, the anodic oxidation and anodized aluminum oxide removal processes were alternately repeated 5 times so that the holes became wide at the top and narrow at the bottom. The nanostructured anodic aluminum oxide (AAO) layer was then electroformed to obtain a nickel mold with a nanoconical cavity array structure. The nickel mold with a nanoconical cavity array structure was used as a master mold to perform thermal embossing on a PC substrate. Figure 1 illustrates the hot embossing process. A hot stamping machine was used to heat and press the nanostructured nickel mold on the surface of the PC substrate for several minutes. After cooling to room temperature, the PC substrate is demolded from the master mold to obtain a PC substrate with a nanocone array structure.

Sputtering was used to form gold nanospheres on top of the nanocones. The PC substrate was rinsed with deionized water before use to remove the fine dust particles on the surface, and then nitrogen will be used to remove the moisture on the substrate surface to reduce the defects caused by fine dust during sputtering. The sputtering process was performed using a DC magnetron sputtering system (Cressington 108 sputtering coater). The sputtering parameters were set as follows: the sputtering duration was 200 s, the sputtering current was 30 mA, and the pressure was kept constant at 0.02 mbar. Due to the self-aggregation characteristics of nano-scale metal materials, the sputtered gold nanoparticles tend to self-aggregate at the tips of the nanocones and form gold nanospheres at the tips of each nanocone (as shown in Figure 1 and Figure 3), resulting in a uniform and closely packed array of gold nanospheres. Since the EC-SERS active substrate can be batch produced by hot embossing and sputtering deposition, with this fabrication technique, we can fabricate disposable, low cost, and highly sensitive EC-SERS substrates with equal quality.

According to our previous work [27], the largest area that could be realized avoiding defects by implementing hot-embossing plus sputtering process method is 120 × 80 mm. The most important step is to produce large-area AAO nanostructures by anodizing. During anodizing, if the surface electric field is unevenly distributed, it will cause uneven oxidation speed and uneven size distribution of nanostructures. Therefore, it is necessary to avoid boundary effects and ensure uniform electric field distribution. In addition, it is necessary to ensure that the pressure is evenly applied to the PC substrate during hot-embossing, otherwise the structure may be different in depth due to uneven force. A vacuum design can be considered to avoid bubbles.

### 2.2. Integrated EC-SERS Chip Fabrication

**Figure 2:**
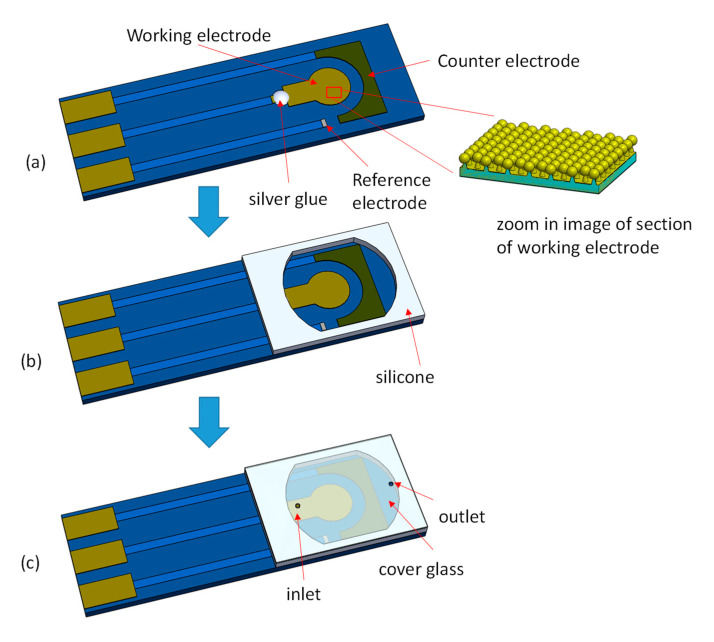
The integrated EC-SERS chip packaging process. (**a**) The EC-SERS active working electrode is placed in the middle of the three-electrode printed circuit board (PCB) substrate, and the working electrode is connected to the PCB substrate with silver glue. (**b**) The reaction area is surrounded with 2 mm high silicone and covered with a cover glass drilled with an inlet hole and a vent hole. (**c**) The packaged integrated EC-SERS chip.

A three-electrode PCB (Figure 2a) was used as a base to combine the fabricated EC-SERS active working electrode and microfluidic reservoir (250 μL volume) into an integrated EC-SERS chip. The detailed dimension of the three-electrode PCB are as follows: Working electrode (WE): 5 mm × 2.5 mm with a circular area diameter 5 mm, Reference electrode (RE): 1.2 mm × 1.4 mm wide (silver/silver chloride), Counter electrode (CE): 13.5 mm × 4 mm (carbon). The integrated EC-SERS chip packaging process is shown in Figure 2. First, the EC-SERS active working electrode was placed in the middle of the three-electrode PCB substrate, and the working electrode was connected to the PCB substrate with silver glue. The reaction area of the three-electrode PCB substrate was then surrounded with 2 mm high silicone. Finally, the reaction area was covered with a cover glass drilled with an inlet hole and a vent hole to complete the packaging of the integrated EC-SERS chip.

### 2.3. Experiments

A scanning electron microscope (SEM, JSM-7800F, JEOL Ltd., Tokyo, Japan) was used to study the surface morphology of the manufactured EC-SERS active working electrode. The EC-SERS performance of the substrate was tested using R6G in NaCl solution (NaCl solution as a supporting electrolyte). A total of 0.25 mL of 10^−7^ M R6G in 0.1 M NaCl solution was injected into the prepared EC-SERS chip for Raman spectrum measurement. The EC-SERS spectra were measured using a microscope Raman spectrometer (Nanofinder 30, Tokyo Instruments, Tokyo, Japan). While measuring, a portable potentiostat (VS1, VidaBio, Taichung, Taiwan) was used for redox scanning and a 632.8 nm HeNe laser was used for excitation. The effective power of the HeNe laser was set to 0.1 mW. The size of the focused laser spot on the sample surface was about 2 μm in diameter, and the integration time of each measurement was set to 10 s. After the measurements were completed, the performance of the EC-SERS chip was characterized by calculating the EC-SERS enhancement factor and the uniformity of the EC-SERS signal on the chip surface.

In addition, we conducted the uric acid EC-SERS spectra measurements. Firstly, we measured uric acid in NaCl solution (NaCl solution as a supporting electrolyte). 4 × 10^−4^ M uric acid in 0.1 M NaCl was injected into the integrated EC-SERS chip for the uric acid EC-SERS spectra measurements. Same as the R6G measurements, the Raman spectrometer was used to collect Raman signals, and the portable potentiostat was used for redox scanning. Secondly, the uric acid EC-SERS spectra were measured in synthetic urine to simulate a more realistic sample. The synthetic urine was prepared according to the literature [8,23,28]. It has considered most of the components of the real urine matrix of healthy people (potential EC-SERS interference). In order to obtain approximately 1 L of synthetic urine, 10 g of urea, 5.2 g of NaCl, 4.5 g of KCl, 4.8 g of sodium phosphate, 0.4 g of citric acid, 0.8 g of creatinine, and 50 mg of albumin were mixed with 900 mL of deionized water. The EC-SERS spectra of various uric acid concentrations in artificial urine were measured. Subsequently, the relationship between the concentration of uric acid in artificial urine and the intensity of EC-SERS was analyzed.

## 3. Results and Discussion

### 3.1. Characterization of the Developed Working Electrode Substrate

**Figure 3:**
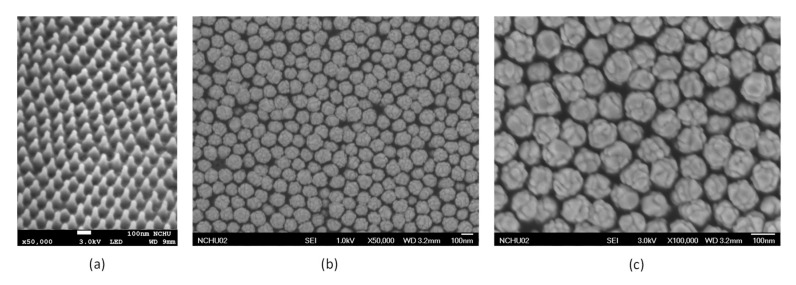
(**a**) SEM image of the PC substrate with nanocone array structure on the substrate surface after the hot embossing process (**b**) The SEM image shows that the gold nanospheres are evenly distributed on the working electrode substrate. (**c**) The nanospheres are displayed at higher magnification for easy observation.

Figure 3a shows the SEM image of the PC substrate after the hot embossing process. The nanocone array structure was formed on the substrate surface and the nanocone array appears evenly distributed. Figure 3b shows the SEM image of the PC substrate after the sputtering deposition of gold. Figure 3c shows the nanospheres at higher magnification for easier to observe. As mentioned previously, the sputtered gold nanoparticles tend to self-aggregate at the tips of the nanocones and form gold nanospheres at the tips of each nanocone. Therefore, the gold nanospheres are evenly distributed closely packed on the working electrode substrate. It is worth mentioning that the diameter of these nanospheres can be controlled by the sputtering time. The diameter of these nanospheres increases with the extension of the sputtering time. Therefore, the diameter of the nanospheres can be fine-tuned by carefully adjusting the sputtering duration, so that the gap between the nanospheres is within a few nanometers.

### 3.2. Simulations

**Figure 4:**
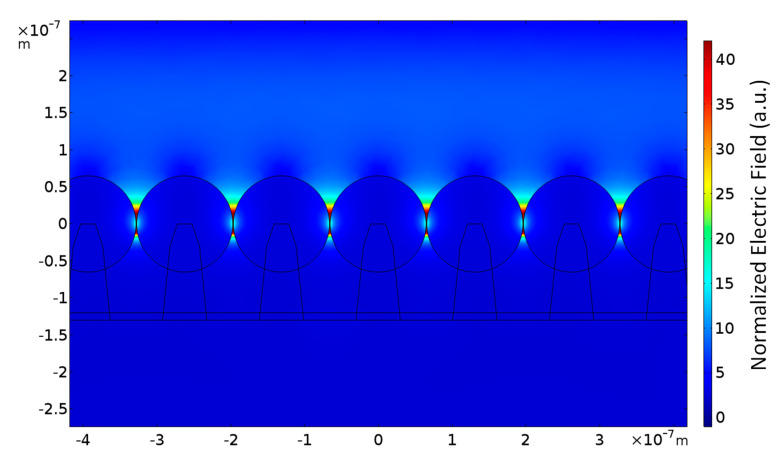
The cross-sectional view of the local electric field distribution around the nanospheres under the incident light wavelength of 632.8 nm is shown here. The normalized electric field in this figure represents the ratio between the electric field intensity excited by the local surface plasmon resonance and the incident electric field intensity. It shows a strong electric field formed between gold nanospheres because of localized surface plasmon resonance of these gold nanospheres.

In order to further understand the influence of the nanocone array substrate decorated with gold nanospheres on the local electric field enhancement, a simulation was carried out. A commercially available finite element method (FEM) software (COMSOL Multiphysics) was used to calculate the local electric field of the gold nanospheres decorated nanocone array structure under the excitation of a 632.8 nm laser source. The average diameter of gold spheres is 133.6 nm and the standard deviation is 8.9 nm. Therefore, the diameter of gold nanospheres was set to 134 nm and the pitch between nanospheres was set to 136 nm in the simulation. The simulated profile view of the local electric field distribution around the nanospheres under the excitation laser wavelength of 632.8 nm is shown in Figure 4. The normalized electric field in this figure is the ratio between the electric field intensity created by the local surface plasmon resonance and the incident electric field intensity. We can see that due to the local surface plasmon resonance of these gold nanospheres, a strong electric field is formed around the gold nanospheres, especially between the nanospheres. These closely packed gold nanospheres can create many evenly distributed strong electric field spots (hot spots) on the chip surface. According to the electromagnetic field enhancement theory [29,30,31], the SERS enhancement factor is proportional to the fourth power of the local electric field intensity. Therefore, these uniformly distributed hot spots can result in a very good enhancement effect for the EC-SERS signal.

### 3.3. EC-SERS Measurements

**Figure 5:**
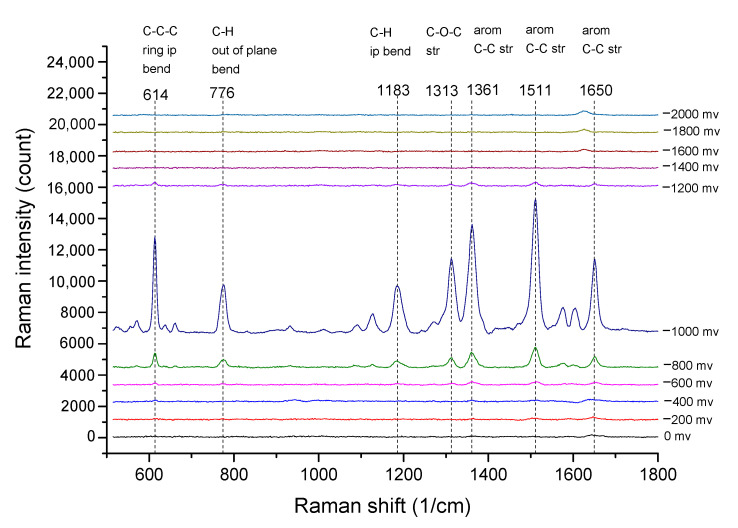
The EC-SERS spectra of R6G at different applied voltage is shown here. The maximum intensity of the Raman signal was reached at −1.0 V.

In order to evaluate the performance of the EC-SERS chip developed in this study and the influence of electrochemical potential on the intensity of Raman signals, first, we use R6G solution to measure the EC-SERS and calculate the Enhancement Factor (EF). To measure the EC-SERS spectrum of R6G, a mixed solution of 10^−7^ M R6G in 0.1 M NaCl supporting electrolyte was injected into the developed EC-SERS chip, and the applied potential was stepping from 0.0 V to −1.0 V with a decrement of 200 mV and recording the spectrum at the same time. Figure 5 shows the reduction stepping scanning EC-SERS spectra of R6G, which were recorded by maintaining potential at each voltage step for 10 s during the acquisition. It can be observed that as the applied potential becomes more negative, the intensity of the Raman signal becomes stronger, reaching the maximum intensity at −1.0 V. When the potential exceeds −1.0 V, the signal strength begins to decrease.

SERS Enhancement Factor (EF) [32] is an important reference for evaluating the effect of SERS enhancement. This parameter can be used to evaluate the enhancement effect of SERS. LeRu et al. [33,34] mentioned that for many applications, a simple question that people are most concerned about is how much more signal can be expected from SERS compared with normal Raman signal under given experimental conditions. When SERS experiments are deal with the analyte in solution, the SERS EF can be naturally defined by normalizing with respect to concentrations. Since the R6G EC-SERS spectrum was measured using the R6G aqueous solution, it would be more meaningful to also use R6G aqueous solution instead of solid R6G to measure the normal Raman spectrum of R6G. We slightly modify its formula to make it suitable for evaluating the EC-SERS enhancement effect. The revised formula is as follows:

(1) $$EF=(I_{\mathrm{EC}\text{-}SERS}/I_{\mathrm{Raman}})\times (C_{\mathrm{Raman}}/C_{\mathrm{EC}\text{-}SERS})$$

where $I_{\mathrm{EC}\text{-}SERS}$ and $I_{\mathrm{Raman}}$ are the EC-SERS spectral peak intensity and the normal Raman spectral peak intensity of the R6G molecule obtained in the experiment (1511 cm^−1^ vibration band peak intensity is used in this study). $C_{\mathrm{EC}\text{-}SERS}$ and $C_{\mathrm{Raman}}$ are the concentrations of R6G aqueous solution irradiated by laser spots under EC-SERS and normal Raman condition, respectively. For normal Raman spectra measurements, the EC-SERS active substrate was replaced with a blank silicon wafer. The R6G solution was filled in the microfluidic reservoir for measurement as for the EC-SERS measurement. Since the focused laser spot diameter and the exposed liquid volume are the same in both EC-SERS and normal Raman condition, therefore the EF can be calculated using Equation (1). The $I_{EC\text{-}SERS}$, $I_{\mathrm{Raman}}$, $C_{EC\text{-}SERS}$, and $C_{\mathrm{Raman}}$ are 8661, 1018, 10^−7^ M, and 10^−1^ M, respectively. The EF was calculated to be 8.5 × 10^6^, which is a very good EF for gold-based SERS substrates [32,35].

**Figure 6:**
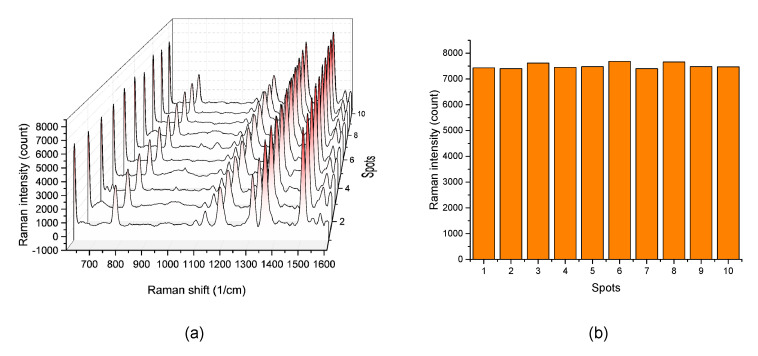
(**a**) EC-SERS spectra of 10^−7^ M R6G at 10 random spots on developed EC-SERS chip. (**b**) The EC-SERS intensities of the Raman peak at 1511 cm^−1^ for 10 random spots on developed EC-SERS chip.

To evaluate the uniformity of the EC-SERS intensity, we used the EC-SERS detection chip developed in this research to measure the 10^−7^ M R6G solution. We measured the EC-SERS signal at 10 random spots on the chip with applied voltage −1.0 V. The measured Raman spectra from 10 random spots is shown in Figure 6a. The EC-SERS intensities of the Raman characteristic peak of 1511 cm^−1^ band from these spots are further plotted in Figure 6b for comparison. The relative standard deviation (RSD) of these EC-SERS intensities was calculated to be 1.41%. Compared with other studies [36,37,38], the RSD value obtained here is very low, which indicates that the SERS enhancement of the developed EC-SERS chip is quite uniform and suite for quantitative measurement. In addition, compared with the SERS chip, the integrated EC-SERS chip can directly detect the analyte solution without waiting for it to dry on the SERS chip. Therefore, it is can avoid the problem of uneven distribution of the molecules to be detected due to the coffee ring effect during the drying process of the solution.

**Figure 7:**
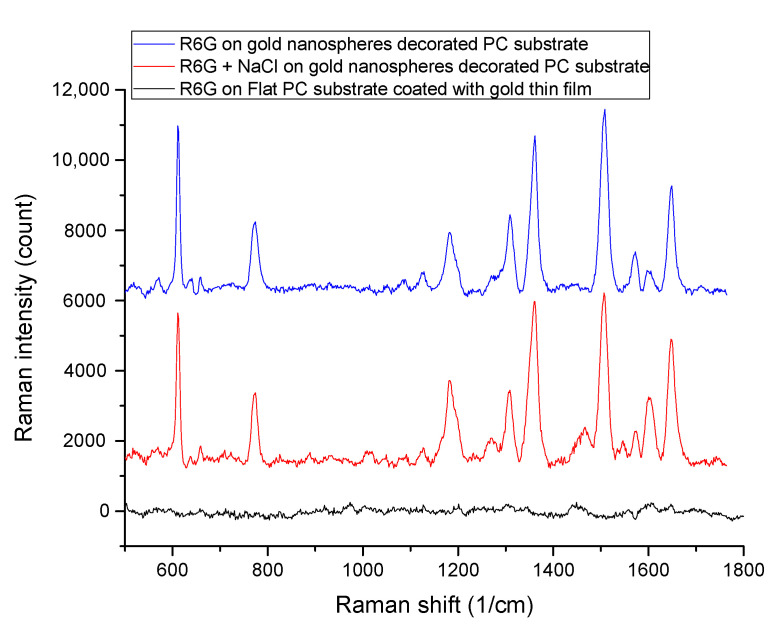
The SERS spectra of 10^−5^ M R6G solution with and without 0.1 M NaCl measured by gold nanospheres decorated PC substrate, and the SERS spectra of 10^−5^ M R6G solution on a thin gold film-coated flat PC substrate.

In order to show that the tightly packed gold nanospheres play a major role in the resulting SERS enhancement, here we compared the SERS spectra of 10^−5^ M R6G aqueous solution on our gold nanospheres decorated PC substrate with 10^−5^ M R6G aqueous solution on a gold-thin-film-coated flat PC substrate (gold film thickness is 100 nm). The result is shown in Figure 7. The SERS signal of R6G can be clearly observed on our gold nanospheres decorated PC substrate. In contrast, the R6G SERS signal cannot be observed on the gold thin film coated flat PC substrate. Therefore, we can infer that the tightly packed gold nanospheres greatly enhance the SERS signal. Figure 7 also shows the SERS spectrum of a 10^−5^ M R6G aqueous solution containing 0.1 M NaCl measured by using our gold nanospheres decorated PC substrate. By comparing the SERS spectra of R6G solutions with and without NaCl, we can see that NaCl has little effect on SERS signal intensity. We believe that since our gold nanospheres were fixed in place from the beginning, there was no other gold nanoparticle aggregation due to the presence of sodium chloride electrolyte. From these results, we can conclude that the resulting SERS enhancement is mainly due to the tightly packed gold nanospheres.

**Figure 8:**
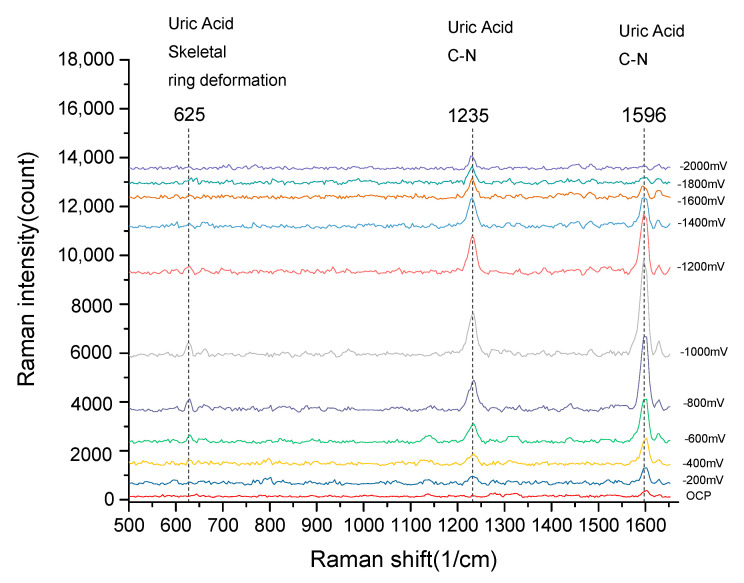
The EC-SERS spectra of uric acid. The uric acid EC-SERS spectra were recorded by stepping the applied potential from 0.0 to −2.0 V in 200 mV decrements.

The developed EC-SERS chip was then used to measure the EC-SERS spectra of uric acid solution. The analyte solution used for uric acid EC-SERS spectra measurement was 10^−4^ M uric acid in 0.1 M NaCl supporting electrolyte. The analyte was then injected into the EC-SERS chip for EC-SERS spectra measurement. Same as the R6G EC-SERS spectra measurement procedure, the uric acid EC-SERS spectra were recorded by stepping the applied potential from 0.0 to −2.0 V in 200 mV decrements. Figure 8 shows the collected EC-SERS spectra of uric acid. The SERS characteristic peaks of uric acid are clearly observed from the spectra. The SERS characteristic peaks of uric acid at 625 cm^−1^, 1235 cm^−1^, and 1596 cm^−1^ are uric acid skeletal ring deformation vibration, C-N stretching vibration, and C-N stretching vibration, respectively. Similar to the R6G EC-SERS result, the potential-dependent of the intensity of the SERS peaks was also observed here. The intensity of the SERS peaks increases as the applied potential move toward negative and reached maximum intensity at −1.0 V. The signal intensity decreased as the applied potential move toward move negative than −1.0 V. The potential-dependent result shows that the best EC-SERS spectrum of uric acid can be detected under an applied potential of −1.0 V. Therefore, for all the following experiments, we collected the EC-SERS spectra of different uric acid concentrations under an applied voltage of −1.0 V.

From Figure 8 we can see that the C-N stretching vibration peak of uric acid at 1596 cm^−1^ is the most intense peak among all the EC-SERS peaks of uric acid. It is the most sensitive peak for the measurement of uric acid. Therefore, for the following measurements, the peak intensity at 1596 cm^−1^ of the EC-SERS spectra measured at applied potential −1.0 V was used to study the relation between the concentration of uric acid and the peak intensity of EC-SERS spectra.

**Figure 9:**
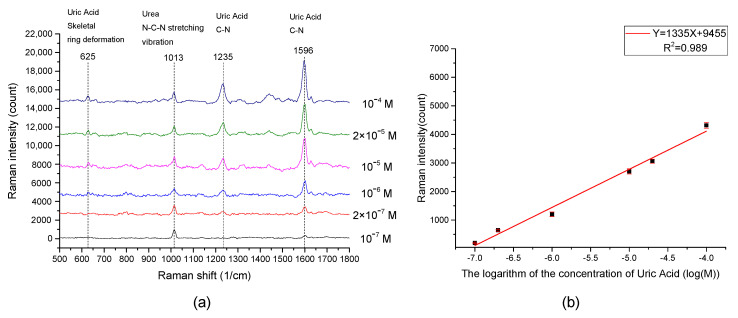
(**a**) The measured EC-SERS spectra of different uric acid concentrations in artificial urine at applied potential −1.0 V. (**b**) Shows the relationship between the concentration of uric acid and EC-SERS intensity of uric acid @1596 cm^−1^ Raman band. It can be seen that the EC-SERS intensity is proportional to the logarithm of the concentration of uric acid.

**Figure 10:**
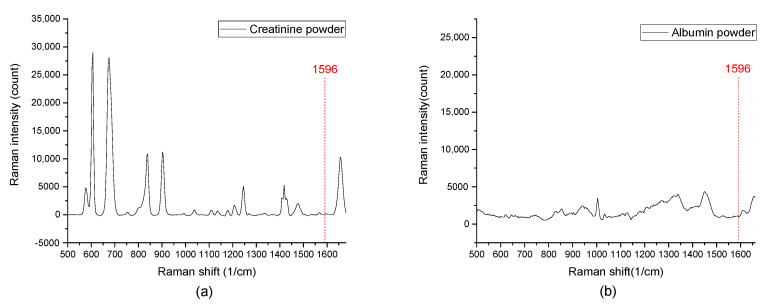
The Raman spectra of pure creatinine and albumin shown in the figure indicate that there is no strong characteristic peak overlap with the 1596 cm^−1^ uric acid peak. (**a**) The Raman spectra of creatinine powder. (**b**) The Raman spectra of albumin powder.

In order to prove that the EC-SERS spectrum of uric acid in urine can be measured, synthetic urine was used as the supporting electrolyte to simulate a more realistic sample. Varying concentrations of uric acid in synthetic urine range from 10^−4^ M to 10^−7^ M were measured as shown in Figure 9a. The characteristic peaks of uric acid at 625 cm^−1^, 1235 cm^−1^, and 1596 cm^−1^ are all can be clearly observed. An additional peak of urea in synthetic urine was observed. Aside from this, there is no other strong interference signal from artificial urine that can affect the measurement of the uric acid EC-SERS spectrum. In addition, the Raman spectra of pure creatinine and albumin showed in the Figure 10 indicate that there is no strong characteristic peak overlap with the 1596 cm^−1^ uric acid peak we used to measure uric acid concentration. Therefore, the results showed that it is feasible to use EC-SERS to detect the concentration of uric acid in synthetic urine.

The peak intensity at 1596 cm^−1^ of the EC-SERS spectra of varying concentrations of uric acid was further extracted to plot against the concentration of uric acid. The result is shown in Figure 9b. There appears to be a logarithmic relationship between the concentration of uric acid and the peak intensity of the EC-SERS intensity. The linear regression of the relationship between the peak intensity at 1596 cm^−1^ of the EC-SERS spectra and the logarithm of the concentration of uric acid in synthetic urine showed that the regression slope was 1335, the intercept was 9455, and the R^2^ was 0.989. An analysis of variance (ANOVA) was performed to assess the linearity. Since the *p* value is very small (*p* = 4.88 × 10^−5^), we can conclude that there is no significant linearity deviation. Accuracy and precision of proposed method were evaluated using three uric acid levels experiments (Table 1). The results showed no significant differences between the expected and calculated concentrations using the proposed method. These results indicate that the EC-SERS signal intensity has a highly linear relationship with the logarithm of the uric acid concentration in synthetic urine, which indicates that the developed EC-SERS chip is suitable for the quantitative detection of uric acid in synthetic urine. Therefore, it is very promising for the developed EC-SERS chip to detect uric acid in real urine samples.

The Limit of Detection (LOD) in this study is defined as the amount of analyte required to generate a signal to obtain a signal-to-noise ratio (S/N) of 3. The noise for the EC-SERS signal was determined by measuring the standard deviation (SD) in the 1596 cm^−1^ region of synthetic urine without uric acid for 10 different spectra, and the SD is 32.5. The LOD of uric acid can then be calculated by using the relation equation in Figure 9b. The resulting LOD is 8.7 × 10^−8^ M. For the LOD of uric acid reported from other studies using EC-SERS technology, Lili Zhao et al. [23] used multilayered Au/Ag as EC-SERS substrate for the quantitative detection of uric acid, and obtained a LOD approximately 1 × 10^−4^ M. Sheila Hernandez et al. [28] measured uric acid in synthetic urine by using electrochemical surface oxidation enhanced Raman scattering. The LOD reported in their study is 12.4 × 10^−6^ M. Barbara L. Goodall et al. [39] used the Ag nanoparticles deposited on silicon as a substrate to perform EC-SERS for detection of uric acid. They reported that the EC-SERS signal of 0.2 mM uric acid in urine simulant can be obtained. As can be seen, compared to the studies above, our EC-SERS chip showed a good LOD of uric acid.

## 4. Conclusions

In summary, we have presented an integrated EC-SERS chip suitable for rapid EC-SERS detection applications. The integrated microfluidic reservoir on the chip makes it easy to use, which is very suitable for rapid detection applications. The proposed EC-SERS active working electrode, which is a nanocone array PC substrate decorated with an evenly distributed and tightly packed array of gold nanospheres, showed good uniformity and can be easily reproduced. According to the R6G EC-SERS experiments, the nanocone array PC substrate combined with evenly distributed and tightly packed gold nanospheres showed a strong enhancement factor up to 8.5 × 10^6^. In addition, the RSD of EC-SERS intensity is as low as 1.41%, indicating that it has good uniformity. Further test results of the detection of uric acid in synthetic urine indicate that the developed EC-SERS chip works well for the quantitative detection of uric acid in synthetic urine. Therefore, it is very promising for the developed EC-SERS chip to quantitatively detect uric acid in real urine samples, and it may be further developed and used in many medical tests, food safety, and biotechnology applications. Further work is underway to extend the use of this chip for portable or on-site applications using miniaturized Raman spectrometer (e.g., AvaRaman system from AVANTES or QE Pro Raman spectrometer designed by OceanInsight, Mira M-1 portable Raman spectrometer of Metrohm or equivalent). We will conduct more quantitative analysis of uric acid using real human urine samples. We will also try to vary the nanocone dimension, the spacing between each nanocone, and the gold nanospheres diameter to obtain an optimum SERS performance.

## Figures and Tables

**Table 1:** Accuracy and precision of proposed method were evaluated using three uric acid levels experiments.

Reference Value	Found ± s (μM)	Accuracy		Precision
		Recovery (%)	Er (%)	
Level 1 (5 μM)	4.15 ± 0.42	83	−7	Level 1 (5 μM)
Level 2 (50 μM)	48.52 ± 4.71	97	−3	Level 2 (50 μM)
Level 3 (95 μM)	99.75 ± 8.35	105	+5	Level 3 (95 μM)
